# CO_2_ exposure at pressure impacts metabolism and stress responses in the model sulfate-reducing bacterium *Desulfovibrio vulgaris* strain Hildenborough

**DOI:** 10.3389/fmicb.2014.00507

**Published:** 2014-09-25

**Authors:** Michael J. Wilkins, David W. Hoyt, Matthew J. Marshall, Paul A. Alderson, Andrew E. Plymale, L. Meng Markillie, Abby E. Tucker, Eric D. Walter, Bryan E. Linggi, Alice C. Dohnalkova, Ron C. Taylor

**Affiliations:** ^1^Pacific Northwest National Laboratory, Biological Sciences DivisionRichland, WA, USA; ^2^Department of Microbiology, School of Earth Sciences, The Ohio State UniversityColumbus, OH, USA; ^3^Environmental and Molecular Sciences Laboratory, Pacific Northwest National LaboratoryRichland, WA, USA

**Keywords:** *Desulfovibrio vulgaris*, geologic CO_2_ sequestration, RNA-Seq, NMR spectroscopy, microbial stress

## Abstract

Geologic carbon dioxide (CO_2_) sequestration drives physical and geochemical changes in deep subsurface environments that impact indigenous microbial activities. The combined effects of pressurized CO_2_ on a model sulfate-reducing microorganism, *Desulfovibrio vulgaris*, have been assessed using a suite of genomic and kinetic measurements. Novel high-pressure NMR time-series measurements using ^13^C-lactate were used to track *D. vulgaris* metabolism. We identified cessation of respiration at CO_2_ pressures of 10 bar, 25 bar, 50 bar, and 80 bar. Concurrent experiments using N_2_ as the pressurizing phase had no negative effect on microbial respiration, as inferred from reduction of sulfate to sulfide. Complementary pressurized batch incubations and fluorescence microscopy measurements supported NMR observations, and indicated that non-respiring cells were mostly viable at 50 bar CO_2_ for at least 4 h, and at 80 bar CO_2_ for 2 h. The fraction of dead cells increased rapidly after 4 h at 80 bar CO_2_. Transcriptomic (RNA-Seq) measurements on mRNA transcripts from CO_2_-incubated biomass indicated that cells up-regulated the production of certain amino acids (leucine, isoleucine) following CO_2_ exposure at elevated pressures, likely as part of a general stress response. Evidence for other poorly understood stress responses were also identified within RNA-Seq data, suggesting that while pressurized CO_2_ severely limits the growth and respiration of *D. vulgaris* cells, biomass retains intact cell membranes at pressures up to 80 bar CO_2_. Together, these data show that geologic sequestration of CO_2_ may have significant impacts on rates of sulfate reduction in many deep subsurface environments where this metabolism is a key respiratory process.

## Introduction

The injection of CO_2_ gas into deep subsurface environments has emerged as one possible process to reduce anthropogenic greenhouse gas (GHG) emissions to the atmosphere (Benson and Cole, 2008). This technology involves the pumping of CO_2_ into suitable geologic environments such as depleted gas and oil fields, saline aquifers, and basalt formations (Holloway, 2005; McGrail et al., 2006). In many of these environments, elevated pressures and temperatures result in a CO_2_ phase change, from a gaseous to a supercritical state. Within deep subsurface environments, supercritical CO_2_ (scCO_2_) can undergo dissolution and disassociation reactions in formation fluids, resulting in pH decreases between 1.5 and 4 units (Mitchell et al., 2010). This pH change can subsequently cause mineral dissolution (and pH buffering) and re-precipitation reactions; the formation of new carbonate minerals may sequester the injected carbon over extended time periods (Krevor and Lackner, 2011; Qafoku et al., 2014).

A significant fraction of microbial biomass on Earth is present in the deep subsurface (Whitman et al., 1998; Kallmeyer et al., 2012; McMahon and Parnell, 2014), in many terrestrial and oceanic locations that could potentially be used for CO_2_ sequestration (Stevens and McKinley, 1995; Krumholz, 2000; Basso et al., 2009; Edwards et al., 2012). scCO_2_ has been shown to significantly reduce bacterial membrane potential and membrane integrity (Tamburini, 2013), and is commercially used as a microbial sterilizing agent in the food industry (Amanatidou et al., 1999). The injection of scCO_2_ into the subsurface could therefore potentially kill a significant fraction of biomass in the storage formation (White et al., 2006). However, the heterogeneity of the subsurface may provide microenvironments where less harsh conditions can be found; indeed, a field test site in Ketzin, Germany reported active populations of sulfate-reducing bacteria (SRB) and methanogenic Archaea following a CO_2_ injection (Morozova et al., 2010). Similarly, studies in an Australian sandstone aquifer revealed increases in *Comamonadaceae* and *Sphingomonadaceae* following CO_2_ injection (Mu et al., 2014). The activity of such microbial populations in subsurface regions receiving injected CO_2_ has both beneficial and deleterious implications for the efficiency of this process. Biofilm formation might act to block small fractures and pores, preventing upward migration of CO_2_ into overlying aquifers (Mitchell et al., 2009), while microbially-enhanced mineral precipitation could accelerate rates of CO_2_ incorporation into insoluble phases (Mitchell et al., 2010). Conversely, microbial growth around injection wells can cause localized clogging (Morozova et al., 2010), corrosion (Pitonzo et al., 2004), and reductions in injection rates, while the potential for certain microbial strains to metabolize CO_2_ and H_2_ to methane, a more potent GHG than CO_2_, remains poorly understood under relevant conditions.

Although *in situ* microbial activity has been reported following CO_2_ injection, little is known about how CO_2_ injection and possible upward migration of CO_2_ into overlying formations can affect microbial physiology, metabolism, and stress responses. Studies on a range of different microorganisms have reported reductions in growth rates and cell yields associated with increasing CO_2_ exposures, while a combination of increased CO_2_ at high pressures caused large reductions in viable cells (Coyne, 1933; Eklund, 1984; Wu et al., 2010; Schulz et al., 2012). Mitchell et al. (2008) reported that biomass present in a biofilm were more resistant to scCO_2_ than similar cells in a planktonic state, and hypothesized that differences in cell viability might be due to protective effects of extracellular polymeric substances (EPS) that accumulate within a biofilm. However, despite these studies there is currently little understanding of the stress response mechanisms that may be utilized by microbial populations that are suddenly exposed to CO_2_ at elevated pressures. Sulfate reducing bacteria (SRB) inhabit a wide range of diverse ecosystems, under conditions that are frequently considered to be “extreme” for microbial growth, including the deep subsurface in terrestrial and ocean environments (Bale et al., 1997; Sass and Cypionka, 2004; Basso et al., 2005; Klouche et al., 2009). Here we exposed a model subsurface SRB species, *Desulfovibrio vulgaris*, to CO_2_ under a range of pressures, using a novel combination of high-pressure nuclear magnetic resonance spectroscopy (HP-NMR) and RNA-Seq to track both physiological and transcriptional responses to CO_2_ stresses that may occur in deep subsurface injection sites.

## Materials and methods

### Bacterial growth

*Desulfovibrio vulgaris* str. Hildenborough (ATCC 29579) was obtained from the ATCC (Manassas, VA, USA), and grown in a defined freshwater media containing lactate (20 mM) and sulfate (20 mM) as electron donor and acceptor, respectively (Lovley et al., 1993) at pH 7.2. HEPES was added at 50 mM as a buffering agent. Cells were grown under strict anaerobic conditions at 30°C with a 100% N_2_ gas headspace. This strain was used as a representative for heterotrophic SRB in the environment.

### CO_2_ exposure experiments

*D. vulgaris* was grown overnight for 20 h at 30°C to a density of OD_600_ ~ 0.5. Growth culture (50 mL) was harvested in an anaerobic glove box (M Braun, Garching, Germany) via centrifugation at 7000 × g for 10 min, and subsequently re-suspended in 65 ml fresh media to an OD_600_ cell density of ~0.2. For CO_2_ and N_2_ exposure experiments at atmospheric pressure, 160 mL serum bottles were sealed with thick rubber butyl stoppers. The headspace of these bottles was flushed with laboratory-grade CO_2_ or N_2_ for 15 min, before being incubated at 37°C. For exposure studies at elevated pressures, re-suspended biomass was added to a custom-built Parr high-pressure incubation vessel (Parr Instrument Company, Moline, IL, USA) within an anaerobic glove box (M Braun). The sealed pressure vessel was removed from the glove box and connected to a high-pressure syringe pump (Teledyne Isco, Lincoln, NE, USA), and pressurized to a desired pressure using CO_2_ or N_2_ gas. The pressure vessel was incubated at 37°C using a warming sleeve (Parr). Experiments were carried out under both N_2_ or CO_2_ at atmospheric (1 bar), 10 bar, 25 bar, 50 bar, and 80 bar pressures. pH was monitored during experiments via an *in situ* high pressure pH probe (Endress & Hauser, Greenwood, IN, USA). All experiments were performed in triplicate.

### Sulfide and cell growth measurements

During incubation experiments, samples were removed from the pressure vessel via a sampling dip tube, while maintaining pressurized conditions. Samples (1 mL) were removed at 0, 1, 2, 3, and 4 h following pressurization and analyzed for aqueous sulfide measurements using the Methylene Blue assay (Hach, Loveland, CO, USA). Sulfide concentrations were measured at 665 nm using a Shimadzu Biospec-1601 spectrophotometer (Shimadzu, Columbia, MD, USA) and absorbance intensities were compared against a standard curve generated from known sulfide concentrations. Cell densities were also measured using the Shimadzu Biospec-1601 spectrophotometer at 600 nm. Estimates of live cells were obtained using a Molecular Probes Live/Dead BacLight Bacterial Viability Kit, according to manufacturer's instructions (Life Technologies, Grand Island, NY, USA). Cell counts were performed using a Nikon 720 Light Microscope with an attached Nikon Super High Pressure mercury lamp, and a fluorescence cube. The results from eight randomized field of view counts were averaged and the Dead/Live ratio determined.

### Transmission electron microscopy

To image CO_2_-impacted cells, a 5 μ L drop of planktonic *D. vulgaris* cell suspension was applied to a 100 mesh Cu grid covered with formvar support film sputtered with carbon (Electron Microscopy Sciences, Hatfield, PA, USA). The cells were allowed to adhere to the grids for 1 min before being blotted with a filter paper, and negatively stained with a 5 uL drop of NANO-W™ negative stain (Nanoprobes, Yaphank, NY, USA). After 30 s, the excess liquid was removed by wicking, and the sample was allowed to air dry. Whole mount samples were examined with a Tecnai T-12 transmission electron microscope (TEM) (FEI Co., Hillsboro, OR, USA) operating at 120 kV with a LaB6 filament. Images were collected digitally with a 2 × 2K Ultrascan 1000 charge-coupled device (Gatan, Pleasanton, CA, USA). Representative images of at least 50 cells per each sample were collected at 10,000× magnification and analyzed to distinguish the presence or absence of cell ultrastructural change.

### EPS extraction and characterization

EPS isolation from *D. vulgaris* biomass was performed using a modified ethylenediaminetetraacetic acid (EDTA) treatment designed to extract EPS from biofilm communities (Eboigbodin and Biggs, 2008; Cao et al., 2011). Samples were collected at 0 and 4 h time points at 50 bar CO_2_ pressure. Biomass from 50 mL of culture was centrifuged at 5000 × g for 20 min at 4°C. The supernatant was discarded and the pellet was resuspended with 10 mL of sterilized 0.9% NaCl solution and centrifuged at 5000 × g for 20 min at 4°C. The supernatant was collected and defined as a “loosely associated EPS” (laEPS) fraction. The process of washing and laEPS collection was repeated three times and the remaining biomass was resuspended in 1% EDTA in 0.9% NaCl to obtain 5 mg biomass/mL of solution. This mixture was incubated at 4°C for 3 h. After incubation, the mixture was centrifuged at 5000 × g for 20 min at 4°C, and the supernatant collected as a “bound EPS” (bEPS) fraction. The resulting pellet was washed three times by resuspension in 10 mL 0.9% NaCl solution and the supernatant was added to the bEPS fraction. Both EPS fractions were clarified using a 0.22 μm filter and kept at 4°C until concentration. EPS fractions were concentrated and washed using Amicon Ultra Centrifugal Filters (Millipore, Billerica, MA, USA) with a molecular weight cut off of 3000 Da. The EPS fractions were reduced to ~3 mL, and were placed separately into Slide-A-Lyzer Dialysis Cassettes (Thermo Fisher Scientific Inc., Rockford, IL, USA) with a molecular weight cut off of 3500 Da. The dialysis cassettes were placed into a 4 L beaker of ultrapure water and were allowed to spin with a stir bar for 16 h at 4°C. After the overnight dialysis, samples were removed from the cassettes and concentrated to ~1 mL. These samples were flash frozen in liquid N_2_ and lyophilized overnight. For analysis, the lyophilized samples were resuspended in sterile ultrapure water and measured using ATR-FTIR (Attenuated Total Reflectance Fourier Transform Infrared Spectroscopy) using a Bruker Vector 22 FTIR spectrometer (Bruker Optics Inc., Billerica, MA, USA). Resulting data was baseline corrected and normalized to the 1045 cm^−1^ sugar peak using IGOR Pro version 6.3.1.2 (WaveMetrics, Inc., Lake Oswego, OR, USA).

### RNA-Seq library preparation and sequencing

For RNA-Seq measurements, biomass was incubated under CO_2_ pressurized conditions (1, 25, 50, and 80 bar) for 1 h at 37°C. Material was quickly sampled, centrifuged at 7000 × g for 10 min at 4°C, and the resulting cell pellet flash frozen in liquid N_2_. RNA was extracted from biomass using Invitrogen TRIzol® reagent, followed by genomic DNA removal and cleaning using the Qiagen RNase-Free DNase Set kit and Qiagen Mini RNeasy™ kit. An Agilent 2100 Bioanalyzer (Agilent Technologies, Inc., Santa Clara, CA, USA) was used to assess the integrity of the RNA samples. Only RNA samples having an RNA Integrity Number between 8 and 10 were selected for downstream analyses. The Applied Biosystems SOLiD™ Total RNA-Seq kit was used to generate the cDNA template library. The SOLiD™ EZ Bead system (Life Technologies, Grand Island, NY, USA) was used to perform emulsion clonal bead amplification to generate bead templates for SOLiD™ platform sequencing. Samples were sequenced on the 5500XL SOLiD™ platform. The 50-base short read sequences produced by the 5500XL SOLiD™ sequencer were mapped in color space using SOLiD™ LifeScope™ software version 2.5 using default parameters against the *D. vulgaris* str. Hildenborough reference genome. A second mapping step removed reads with low quality value scores (<20) to generate output files for downstream analyses. Normalized data as RPKM (Reads per Kilobase per Million mapped) values were used for downstream analyses. Genes were classified as being up- or down-regulated via One-Way ANOVA analysis to identify differences in means across the different conditions. Results were tested via the Tukey HSD test with a Benjamini-Hochberg multiple hypothesis correction to identify differences with *p* < 0.05.

### NMR methods

*D. vulgaris* biomass was prepared as for the batch kinetic experiments, with the following modification: washed cell suspensions were supplemented with 3 mM ^13^C-lactate (99% uniformed labeled ^13^C, Sigma-Aldrich, St. Louis, MO, USA) as an electron and carbon source. An 800 μ L aliquot of cell suspension was added to an appropriate high-pressure NMR tube or NMR rotor in an M Braun anaerobic glove bag (100% N_2_ atmosphere). High pressure tubes were either 5 mm heavy-walled precision pressure valve NMR tubes (Wilmad, Vineland, NJ, USA) for the ^1^H observed NMR experiments (up to 25 bar), or specialized custom-made 7.5 mm NMR rotors described previously (Hoyt et al., 2011; Turcu et al., 2013) for experiments up to 80 bar for the ^13^C NMR experiments. Experiments were carried similarly as above using laboratory grade N_2_, CO_2_, or Carbon-^13^C dioxide (99%-labeled, Sigma-Aldrich- Isotech, Miamisburg, OH) at atmospheric (1 bar), 5 bar, 10 bar, 15 bar, 25 bar, and 80 bar pressures. All experiments were carried out in duplicate. The sealed pressure vessels once removed from the glove box, were connected to a high-pressure syringe pump system (Teledyne Isco), and pressurized to a desired pressure using CO_2_ or N_2_ gas. The pressure tubes or rotors were incubated at 37°C ± 0.5°C using a Blue-M lab oven (Thermal Product Solutions, White Deer, PA) and equilibrated 15 min after gas was brought to the desired pressure. Rotors were mechanically manipulated in a loading chamber/safety cell as previously described (Turcu et al., 2013). After samples were pressurized to temperature and pressure, they were transferred using a safety cell to the NMR instrument and immediately equilibrated at 37°C in the NMR probe.

^1^H observed NMR data was acquired on a Varian Direct Drive (VNMRS) 600 MHz spectrometer (Agilent Technologies) equipped with a Dell PrecisionT3500 Linux workstation running VNMRJ4.0. The spectrometer system was outfitted with a Varian z-gradient triple resonance HCN probe. A time series of short (every 15 min) 2-D gChsqc spectra (^1^H detect and ^13^C-filtered) were collected with a sweep width of 12 ppm (^1^H) in the direct detect dimension with 2048 complex points and 80 ppm (^13^C) in the indirect dimension with 32 increments and acquisition time of 0.285 s per scan. ^1^H 1-dimensional 1D) NMR spectra were derived from the first increments of these 2-D experiments. The 1D ^1^H NMR spectra were the resultant averaged free-induction decays (FIDs) of 8 scans with a 1 s recycle delay between scans. Data were processed and analyzed using VNMRJ 4.0 software and ^1^H spectra with 2048 complex points were processed with zero filling to digitize 4K points and using a sine-bell squared apodization window prior to Fourier Transform (FT). Spectral peak positions of the lactate and acetate peaks were externally referenced relative to a 0.5 mM 2,2-dimethyl- 2-silapentane-5-sulfonate (DSS) (Chenomx, Edmonton, Alberta, Canada) standard. During *D. vulgaris* growth experiments in CO_2_ or N_2_ at various pressures, protons attached to the C2 methylene and C3 methyl of lactate show ^1^H resonances disappearing at ~4.08 and ~1.30 ppm, respectively, while the C2 methyl of acetate shows a ^1^H resonance emerging over time as lactate is oxidized to acetate.

All ^13^C NMR measurements were performed on an Agilent-Varian 300 MHz VNMRS spectrometer (H0 = 7.05 T) at 75.44 MHz Larmor frequency using a direct polarization Bloch-decay sequence with 31.2 kHz proton decoupling for 300 ms. All ^13^C experiments employed a pulse width of 2 μ s (45° flip angle) and used a relaxation delay of 5 s to acquire 120 transients. Spectra are referenced with respect to TMS via a secondary adamantane standard (37.85 ppm). The ^13^C spectral width was 50 kHz and 15,008 data points were acquired per transient. The ^13^C NMR spectra were zero filled to a Fourier number of 32,000 points and given the equivalent of 40 Hz exponential apodization prior to FT. ^13^C resonances for lactate were observed at 185, 74, and 28 ppm for the C1, C2, and C3 carbons, respectively, and for acetate at 184 and 30 ppm for the C1 and C2 carbons, respectively.

## Results

In this study, metabolic and physiological indicators were monitored during exposure of an actively growing SRB model organism, *D. vulgaris*, to CO_2_ over a range of environmentally relevant pressures. Using a high-pressure batch incubation reactor, biomass was incubated under CO_2_ at atmospheric, 10 bar, 25 bar, 50 bar, and 80 bar pressures at 37°C, with samples recovered for kinetic, microscopic, and transcriptomic analyses. High-pressure NMR techniques were applied concurrently to measure microbial oxidation of lactate under similar pressure and temperature conditions.

**Figure 1:**
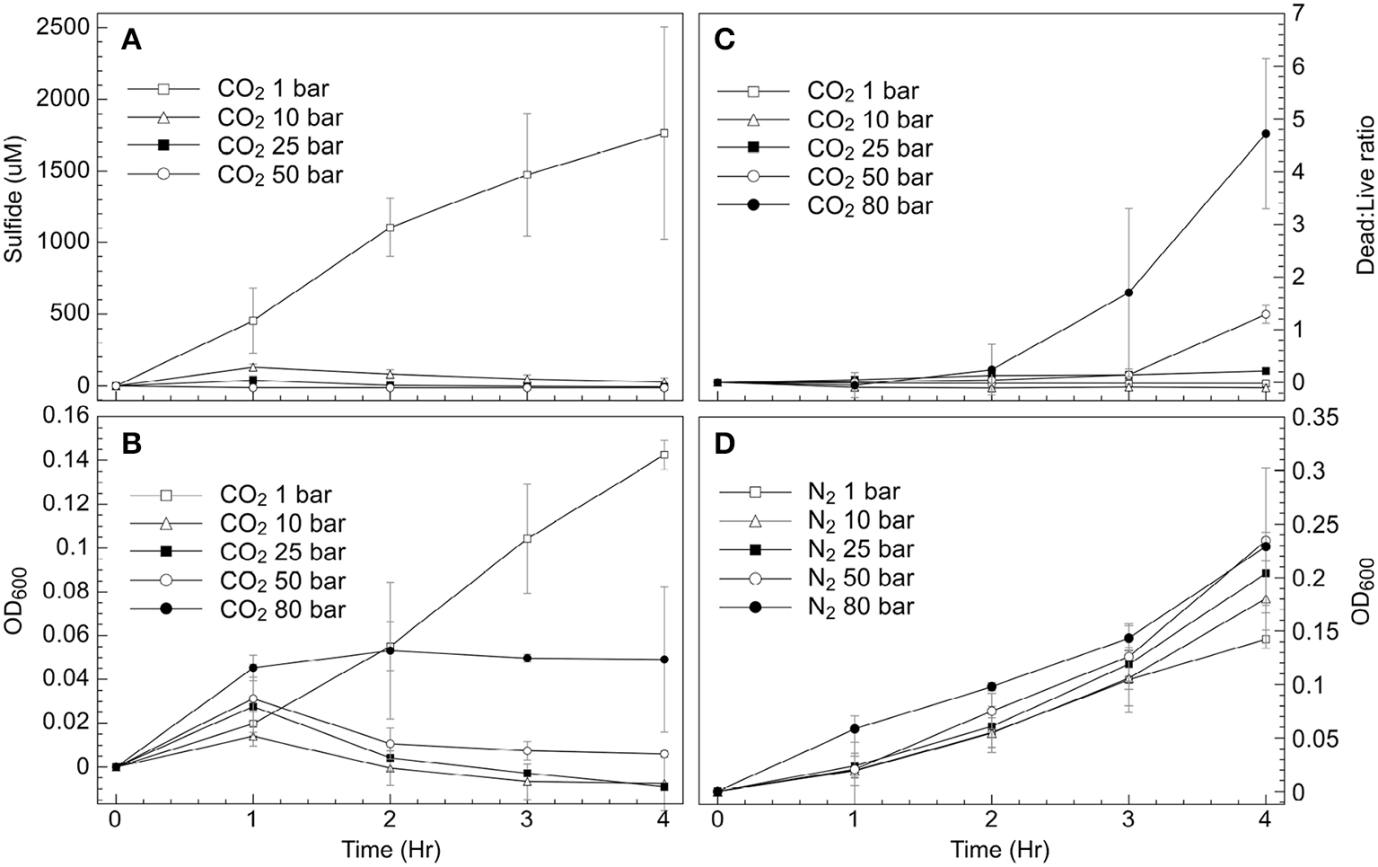
**Exposure of *D. vulgaris* biomass to CO_2_ or N_2_ at a series of pressures**. Values show changes from measurements made at *T* = 0 **(A)** Sulfide concentrations under CO_2_; **(B)** Optical density (OD_600_) under CO_2_ headspace; **(C)** Live:Dead cell ratios under CO_2_; **(D)** Optical density (OD_600_) under N_2_ headspace.

**Figure 2:**
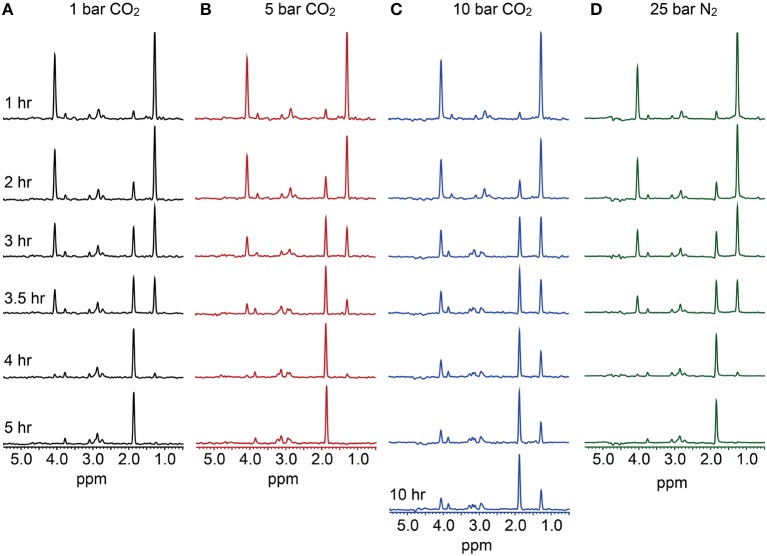
**Carbon (CO_2_ as the pressurizing phase) and proton (N_2_ as the pressurizing phase) NMR data**. Oxidation of ^13^C-3-lactate to acetate was monitored via the disappearance of lactate peaks at 4.04 and 1.26 ppm, which represent the lactate C-H and C-H_3_ bonds, respectively. Concurrent to this, the peak appearing at 1.84 ppm represents the acetate CH_3_ bond. Data indicates that at 1 bar CO_2_ **(A)**, 5 bar CO_2_ **(B)**, and 25 bar N_2_ **(D)**, complete conversion of lactate to acetate occurs over a 4–5 h. period. At 10 bar CO_2_ **(C)** however, incomplete lactate oxidation is observed, supporting observations made in high-pressure CO_2_ batch experiments.

Despite the use of HEPES-buffered media, incubation under CO_2_ (≥5 bar) resulted in pH decreases over the 4 h incubation period from 7.2 to values ranging from 5.5 to 6.5. Atmospheric pressure incubations at pH 6 (N_2_ headspace) indicated that the decrease in media pH had little effect on cell growth or sulfide production (data not shown). CO_2_ exposure had a dramatic effect on cell respiration however, as inferred from sulfide measurements, cell density measurements (Figures 1A,B), and time-course NMR data collection (Figure 2).

Sulfide generation was greatly reduced at CO_2_ pressures above atmosphere (1 bar), with any S^2−^ increases only observed over the first hour of exposure to 10, 25, 50, or 80 bar CO_2_. By contrast, sulfide was consistently generated over the 4-h measurement period under CO_2_ at atmospheric pressure (Figure 1A). These trends were mirrored when measuring cell biomass increases via OD_600_ readings (Figure 1B), with consistent increases in cell density observed at atmospheric pressure under a CO_2_ headspace. At elevated CO_2_ pressures however (≥10 bar), small increases in cell density were generally only observed during the first hour (Figure 1B), with cessation of growth at later time periods. No additional cell growth was observed in these experiments over longer time periods (~48 h) (data not shown). Biomass samples were also recovered at each time point and stained for cell-viability measurements using live-dead cell staining techniques. These data indicated that little cell death occurred at 10 bar and 25 bar CO_2_ pressures during the 4 h incubation period (Figure 1C). Similar trends were initially observed at 50 bar and 80 bar CO_2_. Cell viability began to decrease in these samples at later time points (>3 h), with the greatest proportion of cells with compromised membranes measured after 4 h incubation at 80 bar CO_2_ (Figure 1C). To decouple the effects of elevated pressure and CO_2_ stress, parallel incubations were carried out using N_2_ gas as the pressurizing phase rather than CO_2_. These experiments were carried out at pH 6 to mirror the pH change associated with CO_2_ exposure. Results from these incubations demonstrated that pressure alone did not impact *D. vulgaris* activity. The growth rate in cultures exposed to 80 bar N_2_ (μ = 0.19 h^−1^) was even greater than the growth rate measured in cultures exposed to 1 bar CO_2_ (μ = 0.13 h^−1^) during the 4-h incubation period (Figures 1A,D).

13-Carbon-NMR was used to track the oxidation of ^13^C-3-lactate, and formation of ^13^C-2-acetate by *D. vulgaris* across a range of CO_2_ pressures at 37°C. At 10 bar CO_2_, incomplete oxidation of lactate was observed, indicating inhibition of microbial respiration and supporting observations made in batch reactions (Figure 2). Similar observations were made via NMR at pressures of 25, 50, and 80 bar CO_2_. However, the complete conversion of lactate to acetate was observed at both 1 and 5 bar CO_2_ over similar reaction times, suggesting that some exposure to increased CO_2_ pressures could be tolerated by *D. vulgaris* (Figure 2). In contrast, 1-H proton NMR measurements revealed complete lactate oxidation to acetate by *D. vulgaris* under 25 bar N_2_. These data support batch incubation results that indicated no negative effects of pressure alone on growth rate (Figure 2).

**Figure 3:**
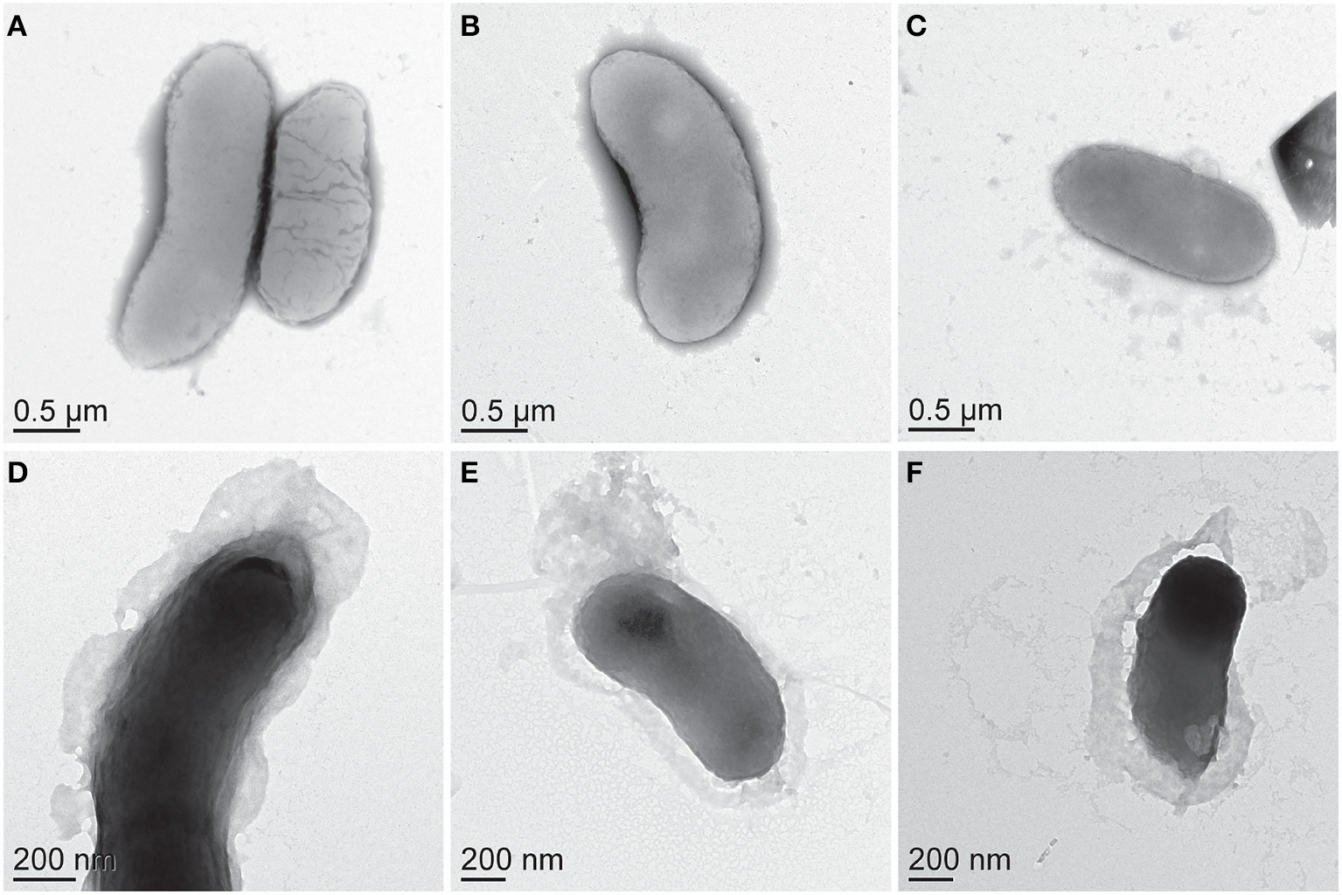
**Negatively stained, whole mount TEM images of *D. vulgaris* biomass (A–C) before, and (D–F) after 4 h exposure to CO_2_ at 50 bar**.

**Figure 4:**
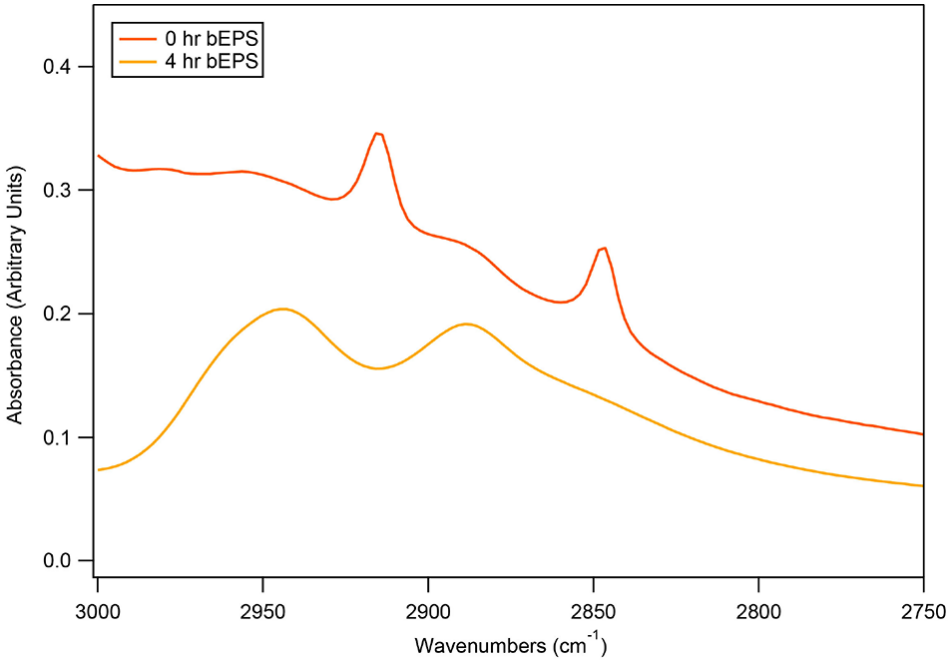
**Comparisons of bEPS chemistry produced by *D. vulgaris* biomass after 0 or 4 h exposure to 50 bar of CO_2_**. EPS was extracted and compared using ATR-FTIR spectroscopy. Ten-minute scans were obtained, baseline corrected, and normalized to 1045 cm^−1^ sugar peak.

Biomass samples taken at 0 h and 4 h time points during 50 bar CO_2_ exposure were imaged using TEM. Initial TEM images showed healthy *D. vulgaris* cells at the beginning of the experiment (Figures 3A–C). After 4 h incubation under 50 bar CO_2_, there was visual evidence for compromised cells, as observed by increasingly shriveled cell membranes (Figures 3D–F). While there are sometimes artifacts from negative strain preparations, here the images may indicate the secretion of large quantities of EPS around cells following 50 bar CO_2_ incubations (Figures 3D–F). To investigate the EPS chemistry, we modified a biofilm EPS extraction protocol to isolate two “operational” EPS fractions, laEPS and bEPS, from the 0 and 4 h time points at 50 bar CO_2_ for chemical characterization. EPS yields were low from all samples. This was not unexpected since biomass in the high pressure batch reactor were generally planktonic. Interestingly, ATR-FTIR spectroscopy did indicate chemical changes in lipid chemistry of the bEPS (EDTA-extractable) fraction after 4 h exposure to CO_2_ (Figure 4). Predominant peaks representing CH_2_- stretches of lipids were observed at 2846 and 2915 wavenumber (cm^−1^) in the 0 h exposure. After the 4 h CO_2_ exposure, these peaks were replaced with CH_3_- lipid peaks at 2888 and 2944 wavenumber (cm^−1^). No chemical differences could be conclusively identified within other classes of biomolecules (proteins, polysaccharides, nucleic acids) in the EPS fractions using ATR-FTIR spectroscopy (not shown).

**Figure 5:**
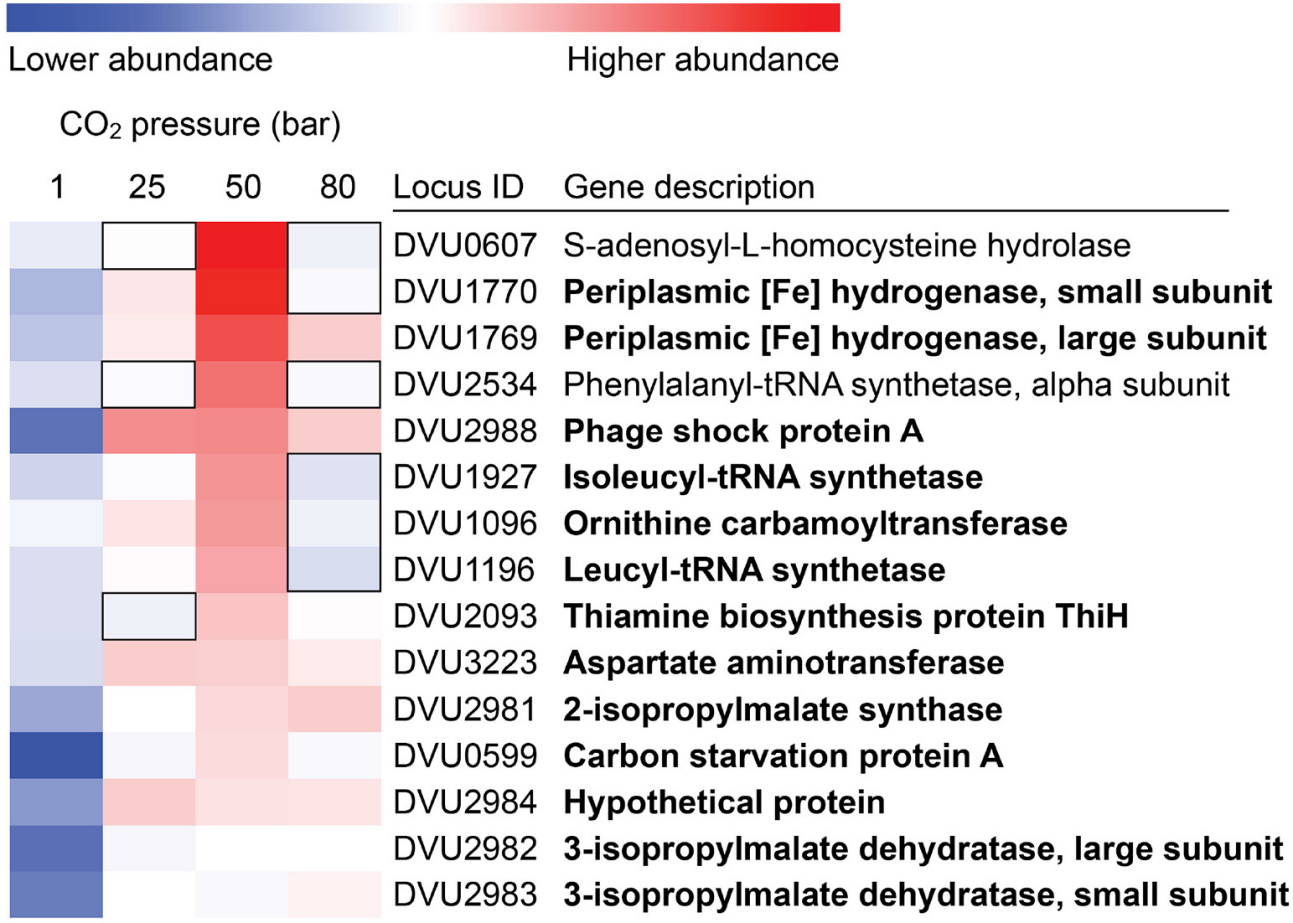
**Expression profiles for up-regulated mRNA gene transcripts under CO_2_ pressurized conditions (25, 50, and 80 bar), relative to 1 bar CO_2_**. Bolded gene descriptions show genes that were up-regulated (*p* < 0.05) under multiple conditions. Conversely, boxed areas show genes that had no statistically significant changes under a certain condition.

Coupled to these physiological measurements, RNA was extracted from *D. vulgaris* biomass that had been exposed to CO_2_ at atmospheric pressure, 25 bar, 50 bar, and 80 bar. Duplicate sets of biomass were generated for each condition. Following conversion of the extracted RNA to cDNA, this material was sequenced using the SOLiD platform. Sequenced reads were mapped to the *D. vulgaris* genome to identify gene expression patterns characteristic of a stress response. Despite the measurement of only limited metabolic activity at elevated pressures, shifts in transcript abundance were observed across these conditions, indicating that cells were responding to CO_2_ stresses (Supplementary Material). Genes associated with chemotaxis (flagella subunits, methyl-accepting chemotaxis proteins) were clearly up-regulated across all CO_2_ exposures, suggesting that cells were attempting to find more suitable environmental conditions (Table 1). However, more genes associated with two-component environmental sensing systems were down-regulated across the same set of conditions (Table 1). The regulation and expression of certain amino acids appears to play a key role in the *D. vulgaris* stress response to pressurized CO_2_ conditions; relative to abundances at atmospheric pressure, transcripts for leucyl and isoleucyl-tRNA synthetases (DVU1196; DVU1927) were both up-regulated at 25 bar and 50 bar CO_2_ pressures, while expression of a tRNA modification GTPase (TrmE; DVU1079) exhibited a similar pattern. Further evidence for a significant amino-acid based response to these stresses was inferred from genes associated with leucine biosynthesis. In addition to the leucyl-tRNA synthetase, transcripts from a 2-isopropylmalate synthase (DVU2981), and both the large and small subunits of a 3-isopropylmalate dehydratase (DVU2982 and DVU2983, respectively) were similarly up-regulated. This pattern was maintained in the 50 bar and 80 bar CO_2_ exposures (Figure 5). Other general stress responses were observed during CO_2_ exposure at 25 bar. The phage shock protein (Psp) system responds to extracytoplasmic stresses, and is induced by dissipation of proton motive force (PMF) in the periplasm (Darwin, 2005). However, although PsPA is the most abundant protein upon induction of the PsP system, little is currently known as to its physiological function. PspA was up-regulated at both 25 bar and 50 bar CO_2_ exposures, possibly suggesting a loss of membrane integrity in some cells. In addition, a periplasmic Fe-hydrogenase that has been shown to play a key role in protecting against oxidative stress (Fournier et al., 2004) was up-regulated over the same conditions. Interestingly, the transcription of carbon starvation protein A (CstA) was up regulated under all pressurized conditions; given that lactate was provided in excess as a carbon and electron source, the *cstA* gene may play a role in a more generalized stress response. The role of hypothetical genes in many cellular functions was also emphasized via transcriptomic results. Across all three pressurized conditions approximately 25% of up- and down-regulated genes were hypothetical, hinting at unknown stress mechanisms and responses to CO_2_ exposure.

**Table 1:** **Numbers of statistically significant up- and down-regulated genes across the CO_2_ exposure conditions**.

**Gene regulation, relative to 1 bar CO_**2**_**	**CO_**2**_ pressure**		
**Number of genes**	**25 bar**	**50 bar**	**80 bar**
Up-regulated	71	84	57
Hypothetical protein	12	14	11
Chemotaxis	10	8	8
Two-component systems	1	1	1
Ribosomes	2	10	1
Down-regulated	40	38	38
Hypothetical protein	16	15	13
Chemotaxis	0	0	0
Two-component systems	4	3	3
Ribosomes	0	0	1

Some functional groups are shown in greater detail.

## Discussion

In this study, we illustrate the negative effects of CO_2_ exposure on microbial activity in the model organism *D. vulgaris*. As CO_2_ injection into subsurface systems occurs, microorganisms are exposed both within the reservoir, and in shallower regions via CO_2_ leakage and migration through cap rock (Harvey et al., 2012). Our findings indicate that even at relatively low pressures (10 bar) some microbial activity is significantly impacted by exposure to CO_2_. Growth of *D. vulgaris* is significantly limited under all pressurized CO_2_ conditions, and respiration (inferred from the measurements of microbial oxidation of lactate, and accumulation of sulfide) is similarly impacted. The rapid growth of *D. vulgaris* under N_2_ pressurized conditions (up to 80 bar) suggests that pressure itself has no negative impact on cell health. Instead, toxic effects from CO_2_ exposure linked to compromised membrane integrity would appear to be responsible for decreases in *D. vulgaris* activity in certain experiments. Kinetic measurements during pressurized CO_2_ exposure experiments suggest that some microbial activity persisted for approximately the first hour, before respiration and cell growth ceased. Despite this cessation of growth under lower pressure conditions (10 bar and 25 bar CO_2_), epifluorescence microscopy observations reveal that cells remained viable. In addition, the continued production of mRNA transcripts at all CO_2_ pressures studied here further supports the observation that *D. vulgaris* cells remain viable during CO_2_ exposures. These results support observations from environmental samples recovered from a saline aquifer in Germany, where SRB biomass remained viable for up to 30 days, despite the cessation of active sulfate reduction following CO_2_ injection (Frerichs et al., 2013).

Electron microscopy images suggest that EPS may be produced in response to CO_2_ exposure, potentially to buffer against deleterious effects. Given that previous studies have at least partly attributed increased resistance to CO_2_ toxicity in biofilm forming bacteria to greater EPS production (Mitchell et al., 2008, 2009; Wu et al., 2010), *D. vulgaris* may utilize a similar approach. ATR-FTIR measurements on EPS extracted from biomass exposed to CO_2_ showed little overall chemical changes in most classes of biomolecules except the lipid region. Notably, bEPS chemical shifts from CH_2_ to CH_3_ stretches may indicate that membrane lipids were being fragmented as a result of the CO_2_ exposure. The relatively short exposure (4 h) of the *D. vulgaris* cells to 50 bar CO_2_ may not have been adequate to completely access chemical changes in EPS composition, or the protective effect of biofilm formation under CO_2_ exposure conditions. In contrast, measuring gene expression may provide a better representation of the physiological responses to short-term 50 bar CO_2_ exposure. Gene transcript data indicates that *D. vulgaris* utilizes additional stress responses, including the accumulation of certain amino acids such as leucine. The accumulation of specific amino acids (in addition to other solutes) is a well-recognized osmotic stress response in many bacteria (Csonka, 1989); indeed, the up-regulation of several genes involved in leucine biosynthesis has been reported in response to KCl stress (Mukhopadhyay et al., 2006). Under salt stress conditions, the role of these amino acids is generally to buffer the cytoplasm and prevent hyperosmotic shock; glutamate and proline are two of the more well studied examples (Csonka et al., 1994; Amin et al., 1995). A potential role for leucine/isoleucine in CO_2_ stress response is less clear, although these amino acids may be important components of other buffering or chaperone proteins, such as ferritin (Götze et al., 2014). Alternatively, amino acid production may be a more general stress response in this microorganism, stimulated via multiple factors. Likewise, phage shock proteins have been observed to increase in abundance under a range of stress conditions, including nitrate, heat, salt, and oxygen stresses (Chhabra et al., 2006; Mukhopadhyay et al., 2006, 2007; He et al., 2010). Previous studies with *E. coli* have identified increased bacterial motility in response to low pH (Soutourina et al., 2002). Although these experiments were all performed under similar pH conditions (~pH 6), CO_2_ stress may have induced greater expression of genes characteristic of motility in *D. vulgaris*. However, unlike nitrate stress, there was little evidence of CO_2_ stress resulting in up-regulation of genes associated with energy metabolism (He et al., 2010).

Using a model SRB system, we tested the combinations of stresses associated with deep subsurface CO_2_ injection (elevated CO_2_ concentrations, pH decreases, and high-pressure) to ascertain the impact of CO_2_ injection on the physiology and metabolism of indigenous deep-subsurface bacteria. We observed that high pressures (80 bar N_2_) had less inhibitory effects on cellular growth than CO_2_ exposures at relatively low pressures. Although some of the pressures used in this study are lower than those in deep subsurface systems, these results indicate that upward migration of CO_2_ from storage reservoirs would impact microbiology in shallower overlying materials (Harvey et al., 2012), with implications for carbon cycling and metal fate and transport. In addition, the potential for inhibition of sulfate reduction in the presence of pressurized CO_2_ may help to limit deleterious subsurface processes such as sulfide-induced corrosion and oil field souring in the subsurface. However, it is worth noting that active microbial processes have been observed in CO_2_ sequestration reservoirs (Morozova et al., 2010; Mu et al., 2014), likely catalyzed by microbial species that exhibit greater tolerance for scCO_2_. Given the results of this study, injected CO_2_ in subsurface systems is likely to act as a strong driving force for shifts in microbial community structure. The effects of these changes on ecosystem function are currently unclear. Therefore, having observed laboratory responses to CO_2_ stresses in a model microbial strain (*D. vulgaris*), future work will attempt to understand the mechanisms that indigenous microorganisms use for survival and growth in CO_2_-impacted deep subsurface environments, and the impact of microbial persistence on system biogeochemistry.

### Conflict of interest statement

The authors declare that the research was conducted in the absence of any commercial or financial relationships that could be construed as a potential conflict of interest.
